# Cytokines and Tumor Metastasis Gene Variants in Oral Cancer and Precancer in Puerto Rico

**DOI:** 10.1371/journal.pone.0079187

**Published:** 2013-11-20

**Authors:** Esther Erdei, Li Luo, Huiping Sheng, Erika Maestas, Kirsten A. M. White, Amanda Mackey, Yan Dong, Marianne Berwick, Douglas E. Morse

**Affiliations:** 1 Molecular Epidemiology Laboratory, Division of Epidemiology and Biostatistics, Department of Internal Medicine, University of New Mexico Health Sciences Center, School of Medicine, Albuquerque, New Mexico, United States of America; 2 University of New Mexico Cancer Center, Cancer Epidemiology and Cancer Prevention Program, University of New Mexico Health Sciences Center, Albuquerque, New Mexico, United States of America; 3 Spectrum Health, Grand Rapids, Michigan, United States of America; 4 Undergraduate Medical Education Program, University of New Mexico Health Sciences Center, School of Medicine, Albuquerque, New Mexico, United States of America; 5 Molecular Biology PhD Program, Princeton University, Princeton, New Jersey, United States of America; 6 Graduate Program in Statistics, Department of Mathematics and Statistics, University of New Mexico, Albuquerque, New Mexico, United States of America; 7 Department of Epidemiology and Health Promotion, New York University, College of Dentistry, New York City, New York, United States of America; Istituto Superiore di Sanità, Italy

## Abstract

**Objectives:**

A cross-sectional epidemiological study explored genetic susceptibility to oral precancer and cancer in Puerto Rico (PR).

**Materials and Methods:**

Three hundred three individuals with a benign oral condition, oral precancer (oral epithelial hyperplasia/hyperkeratosis, oral epithelial dysplasia), or oral squamous cell carcinoma (SCCA) were identified via PR pathology laboratories. A standardized, structured questionnaire obtained information on epidemiological variables; buccal cells were collected for genetic analysis. Genotyping was performed using Taqman® assays. Allelic frequencies of single nucleotide polymorphisms (SNPs) were evaluated in cytokine genes and genes influencing tumor metastasis. Risk estimates for a diagnosis of oral precancer or SCCA while having a variant allele were generated using logistic regression. Adjusted models controlled for age, gender, ancestry, education, smoking and alcohol consumption.

**Results:**

Relative to persons with a benign oral lesion, individuals with homozygous recessive allelic variants of tumor necrosis factor (TNF-α) −238 A/G SNP had a reduced odds of having an oral precancer (OR_adjusted_ = 0.15; 95% CI 0.03–0.70). The transforming growth factor beta-1 (TGFβ-1 −509 C/T) polymorphism was inversely associated with having an oral SCCA among persons homozygous for the recessive variant (OR_crude_ = 0.27; 95% CI 0.09–0.79). The matrix metalloproteinase gene (MMP-1) variant, **rs5854**, was associated with oral SCCA; participants with even one variant allele were more likely to have oral SCCA (OR_adjusted_ = 2.62, 95% CI 1.05–6.53) compared to people with ancestral alleles.

**Conclusion:**

Our exploratory analyses suggest that genetic alterations in immune system genes and genes with metastatic potential are associated with oral precancer and SCCA risk in PR.

## Introduction

Incidence rates for oral and pharyngeal cancer (OPC) in Puerto Rico are among the highest in the Western Hemisphere [1]. In the course of conducting an epidemiological study of oral cancer and precancer in Puerto Rico, we identified a deficit in the detection of oral premalignant lesions and *in situ* cancers on the island relative to that observed on the United States mainland [2], [3]. In addition to socioeconomic and screening disparity issues that are likely responsible for the observed deficit, genetic variants in the immune system and genes that contribute to the metastatic potential of oral cancers may play a role in the excess of oral cancer on the island.

Cytokines are small molecular-weight regulatory proteins of the immune system that are secreted by active immune cells (mostly T cells, especially helper T cells and antigen-presenting cells) and other cell types (epithelial and endothelial cells, fibroblasts, keratinocytes, etc.), which are part of the body's immune surveillance system [4]–[6]. Variability in the coding and non-coding sequences of cytokine genes can strongly affect immune system activity and its capacity to monitor and clear cancerous cells.

Animal models provide evidence indicative of cytokine involvement in the immune system activation process during oral carcinogenesis [7]. In addition, studies conducted on the Indian subcontinent [8], [9], Taiwan [10], Europe [11], and in southern Thailand [12] have reported associations between cytokine gene polymorphisms and oral cancer. To date, however, associations between immune system response gene variants have not been studied in relation to oral precancers or cancers among Puerto Ricans.

Because oral squamous cell carcinomas have a tendency for micrometastases to the head, neck, and upper body [13], [14], and in light of the observed deficit in early-stage oral cancers diagnosed in Puerto Rico, we elected to include in our analyses genetic variants with the potential to influence oral cancer metastasis.

The current paper reports the results of an exploratory analysis of single nucleotide polymorphisms (SNPs) present in T helper Th1 and Th2 cytokine and tumor metastasis genes in relation to the risk of being diagnosed with an oral precancer or squamous cell carcinoma (SCCA) in Puerto Rico.

## Materials and Methods

### Study participants

Details regarding the study and its implementation have been reported previously [15], [16]. Briefly, during the period February 2003 through October 2007, all individuals diagnosed with either a benign oral condition, an oral precancer, or oral SCCA were identified via one of six pathology laboratories located around Puerto Rico. Initially, oral cancer cases were not included in the enrollment; their recruitment began in July 2004. Benign oral conditions (the comparison group) were defined as histopathological diagnoses of a benign adenoma, amalgam tattoo, cyst, fibroma, fibrous histocytoma, granuloma, hemangioma, incontinentia pigmenti, inflammation, benign ulceration, lipoma, mucocele, mucositis, papilloma, Sjoegren syndrome, sialandenitis, or reactive, non-epithelial hyperplasia. Oral precancer was defined as a histopathologic diagnosis of oral hyperkeratosis (HK), oral epithelial hyperplasia (EH), or oral epithelial dysplasia (OED).

Eligible anatomical sites included the lip (mucosa), tongue, gingiva, floor of mouth, palate, and other and unspecified parts of the mouth (ICD-O-3 C00.3-C00.6, C02-C06).

After receiving surgeon-of-record permission to contact an identified subject, we contacted the individual, first via mail, and then via telephone to answer any questions regarding the study and to schedule an in-person interview. At the scheduled in-person interview and after obtaining informed consent, one of two trained, blinded interviewers administered a standardized, structured questionnaire. The interview included questions on sociodemographic (e.g., race/ethnicity, date of birth), lifestyle (e.g., alcohol consumption, tobacco use), and dietary/nutritional (e.g., fruit and vegetable consumption) factors. In addition, buccal cells were obtained from which genetic material was extracted and stored. To ensure sample integrity and an adequate amount of DNA for extensive genotyping, a whole genome amplification process (WGA) was also conducted.

### Genes selected for genotyping

In order to select genes for genotyping, we reviewed pertinent literature and followed a candidate gene approach while considering fundamental genes that can modulate the balance between T helper 1 and 2 (Th1/Th2) responsiveness of T cell activation, immunosuppression [e.g., transforming growth factor beta-1 (TGFβ-1)], and immune system cellular surveillance against tumor cells [interleukins (IL)].

In addition, we included previously published genetic variants implicated in tumor metastasis, e.g., plasminogen activator, or urokinase, known to play a role in extracellular matrix degradation, thereby promoting tumor cell migration and proliferation [17]–[20], and several matrix metalloproteinases (MMPs), including the MMP-1 SNP [21]–[23]. Further, in that genetic variants of the insulin-like growth factor receptor were previously reported to be associated with oral epithelial carcinoma progression [24], it too was included in our genes of interest.

Because there is no centralized, validated genomic information resource for Puerto Rican-specific cytokine or metastasis-promoting gene SNP variant information, we used frequency comparisons of the International Hapmap European ancestry and HapMap African genetic data sets to compare those to frequencies obtained in the Puerto Rican sample after genotyping. In addition, we evaluated the potential contribution of the population substructure to separate the influence of ethnicity, which we previously published as being approximately 70% European, 24.5% African, and 5.7% detectable Native American genetic ancestry [15].

Genotyping was performed using Taqman assays (Applied Biosystem Inc., now part of LifeTechnologies, Grand Island, NY), which enable the use of probes as short as 13 bases. A nonfluorescent quencher was applied to mitigate background fluorescence, and an ABI 7900HT thermocycler and associated software were used to determine allele status.

### Statistical analysis

Descriptive frequency information for each variant allele included in our investigation was determined based upon HapMap data as well as data from Puerto Rican participants. Hardy-Weinberg equilibrium (HWE) testing was carried out to detect unusual frequency distributions in the study population and deviations from HWE noted. We did not include variants that deviated from HWE testing in our genetic modeling.

Allele frequencies of the genetic variants were assessed and aggregated by diagnostic status. Because multiple IL-1 and IL-10 cytokine SNPs were genotyped, we further examined whether haplotype structures of certain variants inherited in linkage disequilibrium could contribute differently to oral precancer or cancer risk. HaploView 4.2 version software (MIT Broad Institute, Cambridge, MA) was used to determine genomic and chromosomal location of the haplotypes [25]. We searched and compared the International HapMap 3 version of the population data to ascertain ethnicity-specific block structures. We retrieved block information for White and African populations, which were similar in structure. The European data appeared to provide the most appropriate structure for Puerto Rican participants, further confirming our previous ancestry estimate analyses in which we found our study population had approximately 70 percent European ancestry [15].

IL-1 and IL-10 haplotypes were reconstructed from the SNP genotype data using PHASE software [26] that employs a Bayesian methodology in which the prior allelic distribution, recorded in a centralized genetic information source, HapMap project, was chosen to approximate the distribution described in our sample [27], [28].

Associations between each haplotype and a diagnosis of oral precancer or cancer were examined by performing haplotype trend regression. The probabilities of having different haplotypes for each individual were incorporated as predictor variables accounting for haplotype phase uncertainty.

We have carried out HWE testing for the entire sample population. For those genetic variants that met HWE (P_HWE_>0.05), we used dominant and recessive disease models to estimate odds ratios (ORs) and their 95% confidence intervals (95% CI) for being diagnosed with an oral cancer or precancer in relation to having a given genetic variant. We also applied additive models to identify relationships between the selected variants of genes important in immune system activity as well as genes influencing OPC metastatic potential, in each instance in relation to disease risk associated with having each additional copy of the variant allele. ORs were adjusted for known risk factors (age, gender, self-reported ancestry, education, smoking and alcohol consumption).

We did not apply multiple comparison procedures to adjust levels of significance for observed measures of association because our primary analytic aim was to explore potential associations between each gene variant and the various disease outcomes under study. In addition, we were concerned that correcting for multiple comparisons would lead to an unacceptable level of type-2 errors.

For selected SNPs that showed an association with oral precancer or cancer in our statistical and genetic modeling, we explored the function of the applicable gene variant using SNPInfo and FuncPred software (http://snpinfo.niehs.nih.gov/snpinfo/guide.htm).

### Ethics Statement

The research protocol and consenting procedures were reviewed and approved by the institutional review boards at New York University and the University of Puerto Rico, Medical Sciences Campus, San Juan, Puerto Rico. In addition, as de-identified biological samples were sent to and DNA extracted at the University of New Mexico Health Sciences Center, School of Medicine, the UNM HSC Institutional Review Board reviewed the molecular component of the research protocol and determined that it did not involve human subjects and was therefore classified as exempt from review.

Written informed consent was obtained, and the consent form stipulated that all participants agreed to provide health questionnaire information and biological samples for research purposes.

## Results

A total of three hundred three individuals were identified as having been diagnosed with a study-eligible oral condition, consented to participate in the study, and provided usable questionnaire data and buccal cells from which usable DNA was extracted.

Table 1 presents summary characteristics of study participants by diagnostic group. While the racial distribution was similar across the diagnostic categories, persons in the oral SCCA group were more likely to be older, male, and less well-educated than persons in the benign and precancer groups. Table 2 summarizes the SNPs evaluated, their frequency distribution based on HapMap data, their frequency distribution in our study, and HWE testing results. Table 3 reports all allele frequencies by diagnostic group for each variant allele in the study.

**Table 1:** Distribution of demographic variables by oral diagnostic category.

Variable		Benign^a^n (%)	Precancer^b^n (%)	SCCA^c^n (%)
**Age** (years)	30–49	52(33.6)	18 (20.9)	7 (11.3)
	50–59	37(23.9)	20 (23.3)	9 (14.5)
	60–69	43 (27.7)	26 (30.2)	18 (29.0)
	≥70	23 (14.8)	22 (25.6)	28 (45.2)
**Gender**	Female	97 (62.6)	46 (53.5)	16 (25.8)
	Male	58 (37.4)	40 (46.5)	46 (74.2)
**Race**	White	100 (64.5)	55 (64.0)	38 (61.3)
	Black	16 (10.3)	9 (10.5)	9 (14.5)
	Others (including Mestiza, Hispanic, Asian, Taino descendent etc.)	39 (25.2)	22 (25.6)	15 (24.2)
**Education (years)**	<12	37 (23.9)	28 (32.6)	34 (54.8)
	12/High School	29(18.7)	15 (17.4)	19 (30.6)
	>12	89(57.4)	43 (50.0)	9 (14.5)
**Total**		**155 (51.2)**	**86 (28.4)**	**62 (20.5)**

a Oral benign conditions.

b Oral HK/EH+OED cases.

c Oral SCCA.

**Table 2:** Genetic variants in cytokine and metastatic potential genes included in the current study.

SNP gene variant	dbSNP ID	Immune system pathways				HapMap/NCBI Frequency Data						Observed Frequency Data			
			Genotypes			Non-Hispanic White Frequencies			African frequencies			Puerto Rican participants			p- value of HWE^a^
IFN-γ −1615 C/T	**rs2069705**	Th1	CC	CT	TT	0.2	0.433	0.367	0.183	0.517	0.300	0.125	0.476	0.399	0.71
IL-12A −980 A/G	**rs2243113**	Th1	AA	AG	GG	0.983	0.017	0	0.983	0.017	0	0.981	0.016	0.003	0.03*
IL-1α −889 C/T	**rs1800587**	Th1	CC	CT	TT	0.417	0.5	0.083	0.301	0.478	0.221	0.488	0.383	0.129	0.03*
IL-1-β +3954 C/T	**rs1143634**	Th1	CC	CT	TT	0.589	0.375	0.036	0.816	0.167	0.017	0.655	0.323	0.022	0.25
IL-1-β −31 C/T	**rs1143627**	Th1	TT	CT	CC	0.407	0.460	0.133	0.417	0.450	0.133	0.277	0.463	0.260	0.21
IL-1-β −511 A/G	**rs16944**	Th1	AA	AG	GG	0.145	0.4	0.455	0.321	0.490	0.189	0.251	0.453	0.296	0.11
IL-2 −384 G/T	**rs2069762**	Th1	GG	GT	TT	0.042	0.5	0.458	0	0	1.000	0.119	0.358	0.522	0.01*
IL-6 −597 A/G	**rs1800797**	Th2	AA	AG	GG	0.2	0.55	0.25	0	0	1.000	0.074	0.363	0.563	0.46
IL-8 −204 C/T	**rs2227306**	Th1	CC	CT	TT	0.476	0.429	0.095	0.867	0.133	0	0.428	0.447	0.125	0.80
IL-8 −251 A/T	**rs4073**	Th1	AA	AT	TT	0.136	0.409	0.455	0.666	0.317	0.017	0.268	0.465	0.267	0.21
TNF-α −238 A/G	**rs361525**	Th1	AA	AG	GG	0	0.087	0.913	0	0.017	0.983	0.006	0.077	0.917	0.12
TNF-α −308 A/G	**rs1800629**	Th1	AA	AG	GG	0	0.433	0.567	0	0.123	0.877	0.010	0.158	0.832	0.72
TNF-β +252 G/T	**rs2239704**	Th1	GG	GT	TT	0.345	0.534	0.121	0.508	0.390	0.102	0.367	0.479	0.154	1.00
IL-10 −1082 A/G	**rs1800896**	Th2	AA	AG	GG	0.217	0.5	0.283	0.450	0.483	0.067	0.447	0.421	0.132	0.26
IL-10 −3575 T/A	**rs1800890**	Th2	AA	AT	TT	0.137	0.49	0.373	0.058	0.288	0.654	0.070	0.366	0.564	0.55
IL-10 −592 A/C	**rs1800872**	Th2	AA	AC	CC	0.087	0.304	0.609	0.203	0.559	0.238	0.096	0.434	0.470	1.00
IL-10 −819 C/T	**rs1800871**	Th2	CC	CT	TT	0.636	0.273	0.091	0.273	0.527	0.200	0.508	0.398	0.094	0.50
IL-13 +98 C/T	**rs20541**	Th2	CC	CT	TT	0.583	0.367	0.05	0.729	0.203	0.068	0.633	0.312	0.055	0.30
IL-13 −1055 C/T	**rs1800925**	Th2	CC	CT	TT	0.653	0.304	0.043	0.250	0.533	0.217	0.584	0.361	0.055	1.00
IL-4 −1098 T/G	**rs2243248**	Th2	GG	GT	TT	0	0.263	0.737	0.033	0.167	0.800	0.010	0.180	0.810	1.00
IL-4 −524 C/T	**rs2243250**	Th2	CC	CT	TT	0.707	0.259	0.034	0.034	0.271	0.695	0.424	0.466	0.110	0.62
IL-5 −745 C/T	**rs2069812**	Th2	CC	CT	TT	0.458	0.423	0.119	0	0.150	0.850	0.293	0.482	0.225	0.57
IL-17A +18 G/A	**rs3819025**	Th17	AA	AG	GG	0.017	0.083	0.9	0	0.117	0.883	0.029	0.276	0.695	0.84
TGF-β1 +29 T/C	**rs1800470**	Immunosuppressive	CC	CT	TT	0.258	0.581	0.161	0	0	1.000	0.238	0.485	0.277	0.65
TGF-β1 −509 C/T	**rs1800469**	Immunosuppressive	CC	CT	TT	0.517	0.345	0.138	0.610	0.339	0.051	0.357	0.453	0.190	0.24
Urokinase C/T (3′UTR)	**rs4065**	-	CC	CT	TT	0.133	0.533	0.330	0.367	0.483	0.150	0.193	0.510	0.297	0.65
IGF-2R A/G	**rs648253**	-	AA	AG	GG	0.265	0.513	0.222	0.354	0.496	0.150	0.190	0.461	0.349	0.35
MMP-1 C/T	**rs5854**	-	CC	CT	TT	0.425	0.451	0.124	0.646	0.327	0.027	0.566	0.376	0.058	0.88

a p-value of the Hardy-Weinberg equilibrium testing for genotypes evaluated in the entire study population.

* marks genotype distributions which did not meet HWE, later removed from analyses.

Abbreviations used for cytokines and metastasis-associated gene variants: IFN-γ – interferon-γ; IL: various interleukins; TNF – tumor-necrosis factors; TGF-β1 – transforming growth factor-beta-1; IGF-2R – insulin-like growth factor 2 receptor; MMP-1 – matrix metalloproteinase 1.

**Table 3:** Allele frequencies of the genetic variants (minor allele) in cytokine genes and genes with metastatic potential by diagnostic status.

dbSNP ID	Benign oral condition^a^	Precancer^b^	SCCA^c^	Precancer & SCCA
**rs2069705**	0.349	0.394	0.375	0.386
**rs2243113**	0.013	0.006	0.018	0.011
**rs1800587**	0.319	0.306	0.330	0.316
**rs1143634**	0.161	0.179	0.214	0.193
**rs1143627**	0.507	0.441	0.527	0.475
**rs16944**	0.497	0.447	0.464	0.454
**rs2069762**	0.313	0.335	0.254	0.304
**rs1800797**	0.280	0.265	0.196	0.238
**rs2227306**	0.352	0.318	0.366	0.337
**rs4073**	0.517	0.447	0.527	0.479
**rs361525**	0.059	0.012	0.054	0.028
**rs1800629**	0.092	0.0647	0.100	0.079
**rs2239704**	0.352	0.429	0.446	0.436
**rs1800896**	0.365	0.347	0.304	0.329
**rs1800890**	0.240	0.317	0.214	0.276
**rs1800872**	0.286	0.335	0.348	0.340
**rs1800871**	0.269	0.294	0.339	0.312
**rs20541**	0.210	0.206	0.232	0.216
**rs1800925**	0.243	0.247	0.218	0.236
**rs2243248**	0.102	0.124	0.0536	0.096
**rs2243250**	0.375	0.306	0.286	0.298
**rs2069812**	0.477	0.494	0.366	0.443
**rs3819025**	0.194	0.159	0.125	0.145
**rs1800470**	0.487	0.506	0.402	0.465
**rs1800469**	0.424	0.435	0.357	0.404
**rs4065**	0.470	0.418	0.445	0.429
**rs648253**	0.408	0.406	0.482	0.436
**rs5854**	0.230	0.229	0.295	0.255

a Oral benign conditions.

b Oral HK/EH+OED cases.

c Oral SCCA.

Table 4 presents results from our logistic regression modeling of cytokine and metastasis-promoting gene variants, as well as IL-1β gene haplotypes, all of which passed HWE. The results presented in the table and described below in terms of functionality prediction are restricted to selected gene variants for which at least one crude or adjusted OR was 2.0+ or conversely, ≤0.5, i.e., biologically meaningful associations, or when the observed association was statistically significant at the 0.05 level. Findings from all additional analyses are presented in Table S1, S2, S3.

**Table 4:** Logistic regression modeling results of selected cytokine and metastasis- promoting gene variants and IL-1β gene haplotypes.

dbSNP ID/Genetic Model	Crude OR† (±95% CI) Precancer vs. benign oral lesion; p-value	Adjusted OR‡ (±95% CI) Precancer vs. benign oral lesion; p-value	Crude OR† (±95% CI) SCCA vs. benign oral lesion; p-value	Adjusted OR‡ (±95% CI) SCCA vs. benign oral lesion; p-value	Crude OR† (±95% CI) Precancer & SCCA vs. benign oral lesion; p-value	Adjusted OR‡ (±95% CI) Precancer & SCCA vs. benign oral lesion; p-value
**rs361525/Additive Model**	**0.22 (0.05–0.93); 0.040**	**0.15 (0.03–0.70); 0.02**	0.91 (0.37–2.24); ns	0.57 (0.13–2.49); ns	0.50 (0.22–1.13); ns	**0.30 (0.11–0.80); 0.02**
**rs361525/Dominant Model**	**0.20 (0.05–0.91); 0.038**	**0.13 (0.03–0.68); 0.01**	1.02 (0.38–2.75); ns	0.60 (0.12–3.08); ns	0.51 (0.21–1.23); ns	**0.31 (0.11–0.89); 0.03**
**rs2243250/Additive Model**	0.73 (0.49–1.10); ns	0.79 (0.50–1.23); ns	0.66 (0.41–1.07); ns	0.56 (0.26–1.21); ns	**0.70 (0.50–1.00); 0.04**	0.77 (0.51–1.17); ns
**rs1800469/Recessive Model**	0.93 (0.49–1.78); ns	0.88 (0.43–1.80); ns	**0.27 (0.09–0.79); 0.02**	0.26 (0.06–1.10); ns	0.64 (0.35–1.16); ns	0.69 (0.35–1.38); ns
**rs1800871/Recessive Model**	1.88 (0.72–4.94 ); ns	1.82 (0.60–5.54); ns	1.91 (0.65–5.63); ns	1.98 (0.32–12.35); ns	1.89 (0.80–4.47); ns	1.72 (0.61–4.83); ns
**rs1800872/Recessive Model**	2.40 (0.91–6.34); ns	2.18 (0.72–6.63); ns	2.57 (0.89–7.46); ns	3.40 (0.60–19.29); ns	**2.47 (1.03–5.91); 0.04**	2.15 (0.76–6.06); ns
**rs1800890/Recessive Model**	1.33 (0.51–3.45); ns	1.43 (0.49–4.17); ns	0.73 (0.19–2.70); ns	0.45 (0.06–3.30); ns	1.08 (0.45–2.59); ns	1.10 (0.39–3.09); ns
**rs1800896/Recessive Model**	1.07 (0.51–2.24);ns	0.98 (0.43–2.21); ns	0.58 (0.21–1.61); ns	**0.19 (0.04–0.94); 0.04**	0.86 (0.44–1.69); ns	0.69 (0.32–1.49); ns
**Haploblock by rs1143627 & rs16944 of IL-1β gene/Additive Model**						
**CA**	0.82 (0.57–1.17); ns	0.86 (0.58–1.27); ns	0.89 (0.58–1.38); ns	1.05 (0.56–1.99); ns	0.85 (0.62–1.16); ns	0.93 (0.64–1.33); ns
**CG**	0.34 (0.04–3.04); ns	0.47 (0.05–4.59); ns	**3.35 (1.06–10.57); 0.04**	2.43 (0.36–16.37); ns	1.64 (0.56–4.8); ns	1.18 (0.73–4.74); ns
**TG**	1.23 (0.86–1.75); ns	1.15 (0.78–1.70); ns	0.95 (0.62–1.45); ns	0.84 (0.43–1.64); ns	1.12 (0.82–1.52); ns	1.04 (0.73–1.50); ns
**rs5854/Dominant Model**	1.13 (0.66–1.93); ns	1.17 (0.65–2.11); ns	1.53 (0.83–2.84); ns	**2.62 (1.05–6.53); 0.04**	1.27 (0.80–2.03 ); ns	1.42 (0.83–2.42); ns

† Unadjusted odds ratio.

‡ Odds ratios adjusted for age, gender, self-reported ethnicity, smoking status, alcohol intake, and fruit & vegetable consumption.

We found evidence of an association between **rs361525**, a TNF-α variant, and having an oral precancerous lesion relative to having been diagnosed with a benign oral abnormality. Using additive genetic modeling, individuals with homozygous recessive allelic variants of the TNF-α −238 A/G SNP had a significantly reduced odds of having an oral precancer (crude OR = 0.22, 95% CI 0.05–0.93) compared to individuals who were heterozygous for the allelic variant. The OR was 0.15 (95% CI 0.03–0.70) after adjusting for age, gender, smoking, alcohol consumption, education, and self-reported ancestry. When we pooled both precancer and squamous cell carcinoma cases, the crude and adjusted odds ratios were 0.50 (95% CI 0.22–1.13) and 0.30, (95% CI 0.11–0.80), respectively. Our findings regarding **rs361525** were similar when dominant genetic modeling was employed.

Functionality evaluation of the TNF-α −238 A/G promoter sequence, **rs361525** SNP revealed that this allelic variant, located at a transcriptional factor-binding site of the promoter region of TNF-α gene, may directly disturb the level, location or timing of TNF-α expression. http://snpinfo.niehs.nih.gov/snpinfo/snpfunc.htm

Additional analyses of cytokine gene variants also suggest a relationship with oral cancer or precancer. Using an additive model, the interleukin-4 (IL-4) −524 C/T SNP, **rs2243250** allelic variant was consistently associated with a relatively weak reduction in the odds of being diagnosed with an oral precancer or oral SCCA; the ORs were statistically significant only when oral precancerous lesions and oral SCCA were combined and compared to participants with benign oral abnormalities (crude OR = 0.70; 95% CI 0.50–1.00).

The functionality prediction of the **rs2243250** allelic variant SNP is similar to that of TNF-α **rs361525** in that the gene variant is considered a potential transcription factor binding site in which changes can profoundly influence production of the Th2 type of cytokine.

The **rs1800469** SNP, a transforming growth factor beta-1 (TGFβ-1 −509 C/T) polymorphism, was negatively associated with having an oral SCCA among persons homozygous for the recessive variant (crude OR = 0.27; 95% CI 0.09–0.79). After adjustment for covariates, the association remained strong, but was only marginally significant (adjusted OR = 0.26, 0.60–1.10).

When we used FuncPred software to evaluate the potential functionality of the TGFβ-1 −509 C/T SNP, it was predicted to be a strong binding site for approximately 50 different transcription factors.

**Figure 1:**
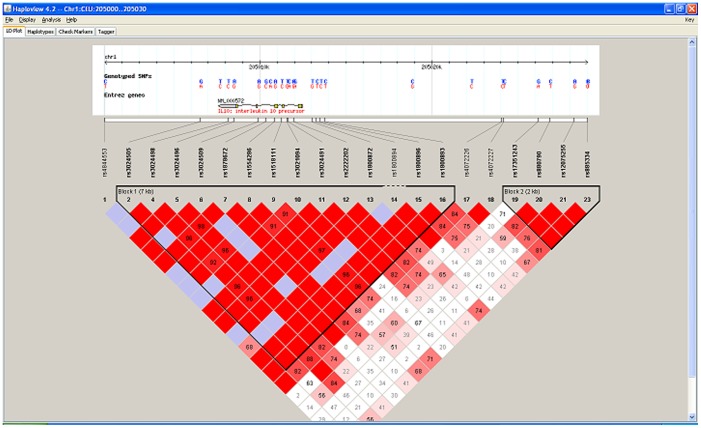
Haplotype analysis of IL-10 cytokine gene variants among Puerto Rican oral precancer and cancer study participants. Footnote: The graph denotes the physical location of the allelic variants on the chromosome and within the examined gene. Bayesian analysis was used to estimate the frequency and structure of the variants among our sample compared to the HapMap Caucasian healthy donor frequencies (see Methods section for more details and references).

Haploblock analysis of the four genotyped IL-10 SNPs resulted in tagging **rs1800896** SNP as the significant contributor to the haplotype structure. When we examined the four polymorphisms independently using a recessive genetic model, the **rs1800896** SNP was negatively associated with oral SCCA (Figure 1, Table 4), while two SNPs, **rs1800871** and **rs1800872**, were positively associated with the risk of having a diagnosis of oral precancer or cancer. ORs for the fourth SNP, **rs1800890**, were highly unstable.

After running functionality prediction on the **rs1800896**, **rs1800871** and **rs1800872** SNPs, they were revealed to be transcription factor binding sites directly influencing production of IL-10, an essential Th2 type of cytokine.

**Figure 2:**
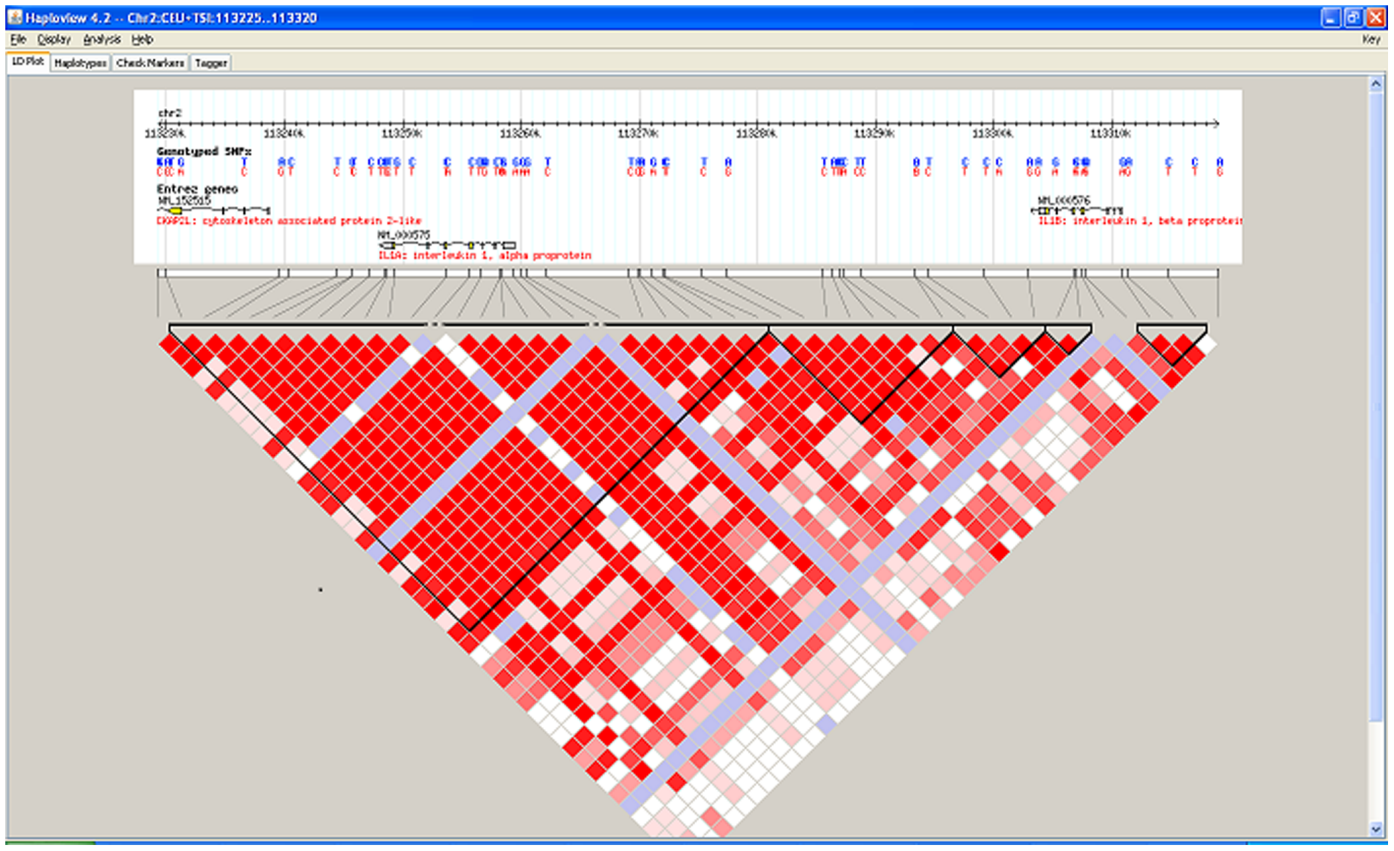
Haplotype analysis of IL-1 cytokine gene variants among Puerto Rican oral precancer and cancer study participants. Footnote: The graph presents actual physical location of the allelic variants on the chromosome and within the IL-1 gene. Bayesian analysis was used to estimate the frequency and structure of the variants among our sample compared to the HapMap Caucasian healthy donor frequencies (see Methods section for more details and references).

We also identified two IL-1 blocks consistent with IL-1α and IL-1β locations. These blocks were similar to recorded ethnic group structures based on updated International HapMap 3 project results (Figure 2). When crude ORs were generated, 2 members of the IL-1β blocks (**rs1143627, rs16944**) as CG haplotype were associated with oral SCCA risk (Table 4.)

In addition to cytokine polymorphisms, we found that among the set of metastasis genes, one crucial genetic variant, **rs5854**, located in the matrix metalloproteinase gene (MMP-1), was associated with oral SCCA risk. Participants who had even one variant allele were more likely to have been diagnosed with an oral SCCA (adjusted OR = 2.62, 95% CI 1.05–6.53) compared to people with ancestral alleles (Table 4).

The **rs5854** SNP was predicted by FuncPred software to be a microRNA binding site. Further, based upon information obtained through the miRBase database (http://www.mirbase.org/index.shtml; University of Manchester, UK), presence of the T variant allows binding of six different miRNAs.

## Discussion

Immune system variants could reflect an overall immune system genetic susceptibility toward cancer development. The current study was based on an *a priori* interest in evaluating pro-inflammatory and anti-inflammatory cytokines as well as metastasis-promoting genes in relation to oral precancer and cancer.

We assessed associations between multiple SNPs in relation to the various disease outcomes of interest. Given the hypothesis-generating nature of the study, we elected to report OR-specific findings from each of our gene- and haplotype-specific analyses whether or not an association was identified and whether or not an observed association was statistically significant (Table 4 and Table S1 through Table S3). By so doing, we provide others with the opportunity to compare their findings to ours. Given the multiple analyses conducted, it is possible that one or another of our findings may have resulted by chance alone. With that caveat in mind, our analysis identified several SNPs that may be related to oral precancer or cancer in the Puerto Rican study population.

Hispanics are underrepresented in oral cancer genetic research despite the fact that some population groups, including Puerto Ricans, have high oral cancer incidence rates [1], [29]–[31]. In the current paper, we present results demonstrating that immune system variation may influence tumor surveillance and immune response through cytokine production. In addition, we found that the matrix metalloproteinase-1 genetic variant, even the presence of only one variant allele, increased the risk of having oral cancer among Puerto Ricans.

It is important to note that although the International Hapmap database reports “Hispanic ethnicity” genotype and allele frequency information for most of the SNPs used in our study, Hapmap data were compiled using genetic material from Mexican donors, which is not necessarily reflective of Puerto Ricans or their genetic ancestry. To enhance accessibility of Puerto Rico specific genetic information, we provide frequency data, stratified by diagnostic group, for each of the variants under study.

Tumor necrosis factor alpha (TNF-α) polymorphisms are frequently noted in oral cancer research [10], [32]–[34]. TNF-α promotes cancer cell apoptosis and tumor-associated killing through the tumor necrosis factor-related apoptosis-inducing ligand (TRAIL) signaling pathway. Nuotomi et al. and Sharma at el. both found that the rs361525 promoter −238 variant increased TNF-α expression and resulted in increased serum production of the cytokine [35], [36]. They also documented increased attraction of neutrophil granulocytes to the lung by TNF-α production. Based upon their findings, one can hypothesize that an increased producer phenotype could be advantageous during oral carcinogenesis.

The same TNF-α SNP, **rs361525**, −238 intronic A allelic variant was previously described as being less frequent in oral cancer cases in Taiwan relative to healthy controls [10], a finding consistent with the protective association we observed among Puerto Rican cases. Notably, the possible protective effect observed in our study was strong, especially as regards precancerous lesions. The similarity in results across disparate populations warrants further, more detailed oral cancer genetic susceptibility research.

Haploblock analysis of the four genotyped Interleukin 10 (IL-10) SNPs revealed that one SNP (**rs1800896**) was negatively associated with oral SCCA, while two other SNPs, **rs1800871** and **rs1800872**, were positively associated with the risk of having a diagnosis of oral precancer or cancer. These results suggest that in future studies it will be prudent to investigate these allelic alterations independently, rather than using a tagging SNP that potentially conceals unknown effects of linked variants and falsely diminishes the effects of IL-10 genetic variants in oral carcinogenesis.

Interleukin1 (IL-1) is one of the strongest T cell activator cytokines. High IL-1 β production is reported to be immunosuppressive in *in vitro* oral squamous cell carcinoma cultures, but the role of different allelic variants on oral cancer risk has not been explored to date. Based on our analysis, the CG haplotype variant of **rs1143627** and **rs16944** SNPs of IL-1β gene was significantly associated with an increased risk of having oral SCCA using an additive disease model.

Interleukin 4 (IL-4) is involved in the regulation of apoptosis and migration of macrophages in numerous cancers. The action of IL-4 during carcinogenesis is possibly linked with the presence and induced cellular activity of tumor-associated macrophages (TAM), which exhibit so-called M2 phenotypes that promote progression and cancer metastasis [37]–[39]. Furthermore, these macrophages enhance matrix remodeling which links immune system cellular activity with metastatic potential, especially through MMP-1 activation [40].

In keeping with our results that suggest a probable protective effect of the **rs2243250** variant against tumor development, it has been reported that in prostate cancer, the IL-4 5′ variant of promoter sequence, **rs2243250** decreases IL-4 activity [41].

The transforming growth factor beta superfamily of cytokines is thought to have complex and contradictory growth effects in several cancers. We found that the **rs1800469** SNP, a transforming growth factor beta-1 (TGFβ-1 −509 C/T) promoter polymorphism, showed protective effects in relation to oral cancer. In addition, the SNP was predicted by FuncPred software and logic minimization to be a strong binding site for approximately 50 different transcription factors. Our findings highlight the need for more oral cancer-oriented, basic immunological studies; perhaps *in vitro* experiments in which this fundamental cytokine may be used for clinical benefit in oral cancer prevention.

After adjusting for covariates, we found that persons with one or more variant allele for the MMP-1 SNP, **rs5854**, had over a 2.5-fold increase in the odds of oral SCCA. Similar to our findings, a previous study involving 96 Chinese patients reported that the presence of MMP-1 homozygous allelic variants in the promoter region of the gene contributed to an increased risk of oral squamous cell carcinoma [42].

MMP-1 proteins, also called interstitial collagenases, break down the extracellular matrix in normal developmental and disease processes. MMPs are frequently noted as contributing factors in cancer metastasis, allowing restructuration of connective tissue surrounding the tumor. The MMP-1 enzyme breaks down types I, II, and III interstitial collagens.

We also provide new information based upon our Puerto Rican oral cancer cases showing that the T allelic variant of the MMP-1 gene potentially promotes several microRNA binding sites. It has been proposed that miRNA sequences can influence not only gene expression but also contribute to methylation patterns and activity changes in some epithelial tumors [43], [44].

MMP-1 has been used as a target of first-generation matrix metalloproteinase inhibitor, first as an adjunct to standard chemotherapy in non-small cell lung carcinoma patients and then in the treatment of HIV-related Kaposi's sarcoma and prostate cancers. Our results indicate genetic susceptibility at the MMP-1 gene that could have potential applicability in oral squamous cell carcinomas; however, based on the National Cancer Institute Clinical Trial information database, MMP-1 inhibition has never been used in oral cancer therapy (http://www.cancer.gov/clinicaltrials/search/results?protocolsearchid=8196085).

Taken together, potential biological links were demonstrated in our analysis; we found that IL-4 variants were present in Puerto Rican participants, and the detected variants can influence tumor infiltrating macrophage activity and their presence in and surrounding the lesions. The tumor infiltrating M2 macrophages promote the matrix remodeling process through increased MMP-1 activation. We identified that even one variant allele of the MMP-1 gene increases the risk of having an oral cancer diagnosis in Puerto Rico. MMP-1 activation enhances the spreading of cancerous cells, thereby contributing to the increased metastatic potential of oral tumors and advanced stage at diagnosis.

Our results not only confirm previous reports, but might suggest molecular and cellular interactions linking genetic variability in immune cell activation and increased metastatic potential among oral cancer cases residing in Puerto Rico. Unfortunately, however, our limited sample size precludes a meaningful formal evaluation of gene-gene or gene-environment interactions.

We note that several potentially interesting SNPs were not detected as influencing oral cancer or precancer risk in our pilot study sample. For example, IL-2 cytokine is also a strong T cell activator [6] and used in several epithelial cancers as adjuvant to chemo- or radiation therapy. However, we did not detect any variants that would show possible immunological potential in this study.

Puerto Ricans are understudied and underrepresented in oral cancer research and prevention efforts. While currently available strategies are likely sufficient to achieve recent World Health Organization goals for reducing oral cancer [45], future prevention measures may ultimately integrate genetic aspects of disease susceptibility. We found evidence among our participants supportive of the possibility that genetic alterations in several essential immune system genes influencing host factors and diminished immune surveillance may contribute to the enhanced risk of oral cancer and precancer in Puerto Rico.

We urge interested colleagues and stakeholders to launch large oral cancer and precancer research efforts serving Hispanic communities disproportionately affected by oral and pharyngeal cancers. Results of those future efforts will likely benefit other ethnic groups as well.

## Supporting Information

Table S1 All SNPs used in genetic disease additive modeling in the Puerto Rican oral precancer and cancer study participants. OR = Odds Ratio. 95% CI = 95% confidence interval. Crude p-value = p value resulted in modeling that estimated unadjusted odds ratios. Adjusted p-value = p-value of the odds ratios adjusted for age, gender, self-reported ethnicity, smoking status, alcohol intake, and fruit & vegetable consumption. Precancer: Oral precancer was defined as a histopathologic diagnosis of oral hyperkeratosis (HK), oral epithelial hyperplasia (EH), or oral epithelial dysplasia (OED). SCCA = squamous cell carcinoma
(XLSX)

Table S2 All SNPs used in genetic disease dominant modeling in the Puerto Rican oral precancer and cancer study participants OR = Odds Ratio. 95% CI = 95% confidence interval. Crude p-value = p value resulted in modeling that estimated unadjusted odds ratios. Adjusted p-value = p-value of the odds ratios adjusted for age, gender, self-reported ethnicity, smoking status, alcohol intake, and fruit & vegetable consumption. Precancer: Oral precancer was defined as a histopathologic diagnosis of oral hyperkeratosis (HK), oral epithelial hyperplasia (EH), or oral epithelial dysplasia (OED). SCCA = squamous cell carcinoma
(XLSX)

Table S3 All SNPs used in genetic disease recessive modeling in the Puerto Rican oral precancer and cancer study participants OR = Odds Ratio. 95% CI = 95% confidence interval. Crude p-value = p value resulted in modeling that estimated unadjusted odds ratios. Adjusted p-value = p-value of the odds ratios adjusted for age, gender, self-reported ethnicity, smoking status, alcohol intake, and fruit & vegetable consumption. Precancer: Oral precancer was defined as a histopathologic diagnosis of oral hyperkeratosis (HK), oral epithelial hyperplasia (EH), or oral epithelial dysplasia (OED). SCCA = squamous cell carcinoma
(XLSX)
